# Promoting fast MR imaging pipeline by full-stack AI

**DOI:** 10.1016/j.isci.2023.108608

**Published:** 2023-12-02

**Authors:** Zhiwen Wang, Bowen Li, Hui Yu, Zhongzhou Zhang, Maosong Ran, Wenjun Xia, Ziyuan Yang, Jingfeng Lu, Hu Chen, Jiliu Zhou, Hongming Shan, Yi Zhang

**Affiliations:** 1 School of Computer Science, Sichuan University, Chengdu, Sichuan, China; 2 School of Cyber Science and Engineering, Sichuan University, Chengdu, Sichuan, China; 3 Institute of Science and Technology for Brain-inspired Intelligence, Fudan University, Shanghai, China

**Keywords:** Medicine, Artificial intelligence, Machine learning

## Abstract

Magnetic resonance imaging (MRI) is a widely used imaging modality in clinics for medical disease diagnosis, staging, and follow-up. Deep learning has been extensively used to accelerate k-space data acquisition, enhance MR image reconstruction, and automate tissue segmentation. However, these three tasks are usually treated as independent tasks and optimized for evaluation by radiologists, thus ignoring the strong dependencies among them; this may be suboptimal for downstream intelligent processing. Here, we present a novel paradigm, full-stack learning (FSL), which can simultaneously solve these three tasks by considering the overall imaging process and leverage the strong dependence among them to further improve each task, significantly boosting the efficiency and efficacy of practical MRI workflows. Experimental results obtained on multiple open MR datasets validate the superiority of FSL over existing state-of-the-art methods on each task. FSL has great potential to optimize the practical workflow of MRI for medical diagnosis and radiotherapy.

## Highlights

• A novel AI paradigm for the full-stack fast MRI imaging
• One network to simultaneously address sampling, reconstruction, and segmentation
• Semantic information can be used to guide the acquisition of k-space raw data
• Significant performance benefits for all tasks validated on multiple MRI datasets

## Graphical abstract

**Figure:**
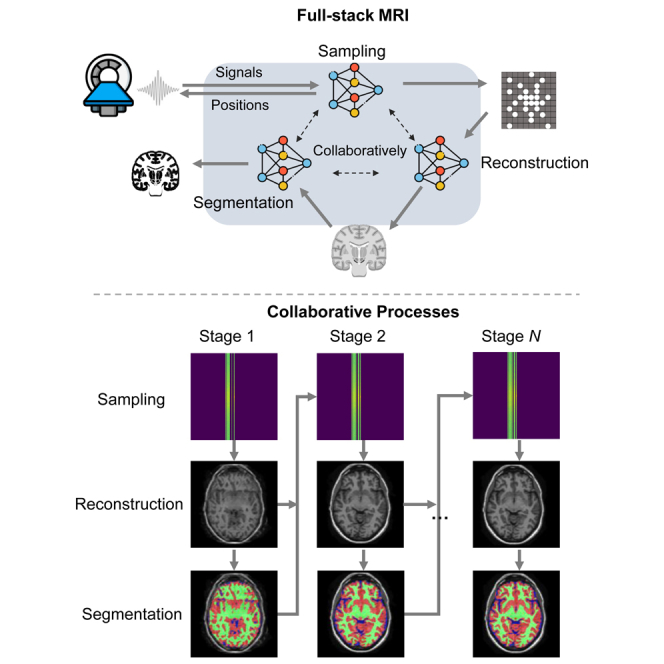

## Introduction

Magnetic resonance imaging (MRI) is a noninvasive diagnostic imaging technique that enables the study of low-contrast soft tissue structures without imposing harmful radiation risks. However, the long acquisition time of MRI results in increased costs, patient discomfort, motion artifacts, and some time-restricted scenarios. Many clinical indicators can be imaged with compressed sensing-based MRI (CS-MRI) to accelerate the imaging process without significantly compromising the image quality or diagnostic accuracy.^1^ In fact, minimizing the amount of required MRI-measured data is a commonly accepted practice, and CS-MRI has been a hot research topic in the medical imaging field for almost two decades.^2^^,^^3^^,^^4^^,^^5^^,^^6^ However, a reduction in the measured data might lead to missed important anatomical information and can introduce artifacts into the reconstructed images, which may adversely jeopardize the subsequent analysis or diagnosis if these problems are not overcome.

Benefitting from rapid developments of both algorithms and computer hardware, significant advances have emerged from intelligent fast imaging^5^^,^^6^^,^^7^ and accurately automated medical image analysis^8^^,^^9^^,^^10^ via deep learning^11^ (DL). Specifically, magnetic resonance (MR) images need to be measured partially and reconstructed by scanners or processed and analyzed by servers. While most images in the past are sampled and reconstructed for human examination visually, in the era of intelligent computing, an increasing number of visual images can simultaneously serve human perception and power machine vision-based intelligent systems. Currently, existing imaging methods are mainly designed for either medical image reconstruction or medical image analysis, leading to fragmented visual processing stages.

Traditionally, various DL reconstruction paradigms have been proposed for imaging pipelines due to their data-driven superiority. These DL frameworks can be categorized into the following categories: (1) CS-MRI,^12^ (2) compressed sampling learning^13^^,^^14^^,^^15^^,^^16^ (CSL), and (3) compressed sensing-based multitask learning^17^^,^^18^^,^^19^^,^^20^ (CS-MTL). DL-based CS-MRI methods form hand-crafted trajectories that are developed to achieve the desired visual fidelity; then, the reconstructed images can be fed into machine analysis tasks. In this way, artifacts and missing measurements can be visually processed in the data or/and image domains, yet data incompatibility between hand-crafted trajectories and scene-specific scans can be encountered, resulting in redundant sampling. Over the past year, we have started to see the development of learnable trajectory methods based on DL, called CSL approaches, which significantly reduce redundancy in such a way that reliable images can be reconstructed. However, the resultant methods formed by both CS-MRI and CSL often suffer from downstream tasks loss, inducing inaccurate medical image analyses and distorted high-level semantics that indicate structure distortion and detail loss in image reconstruction. DL-based CS-MTL paradigm aims to jointly learn reconstruction vision tasks and analysis vision tasks to link this disconnection, and this technique has achieved remarkable improvements. Unfortunately, such a CS-MTL paradigm that is conventionally trained with hand-crafted trajectories, also disconnects reconstruction–analysis vision from the measurement of raw data, thereby incurring fragmented observation and imaging stages for both visions, leading to a high level of risk for MR imaging pipelines. The main motivation of this study is to identify the opportunities and challenges involved in developing a novel full-stack MTL framework for collaborative and scalable modes to achieve improved imaging performance with efficient expression in the data domain for both image reconstruction and analysis vision, and establish a foundation for an MRI pipeline in which both visions are connected for all tasks by big data. Toward that goal, our method of choice is a novel full-stack MTL approach implemented with a novel neural network framework. In addition to the improved performance achieved on all tasks, the implication is clear: if the full-stack collaborative MTL approach performs favorably or comparably to the fragmented processing mechanism, the use of the full-stack paradigm in collaborative manner in the medical imaging pipeline might yield a practical advantage over traditional processing solutions. As the real scanning and imaging processes are nonintegrable, we simulate the full-stack approach to compare various DL methods for three traditional paradigms on fair ground. We stress that our intent is to demonstrate the superiority of full-stack collaborative MTL pipelines over traditional imaging paradigms implemented by mainstream methods instead of developing a specific neural network for improving the performance of specific tasks.

With the rapid development of MTL techniques, joint reconstruction–analysis vision tasks have recently produced great results for image processing pipelines. At present, MTL-based methods only start from reconstruction vison tasks and extend to machine vison tasks with a preset observation. In this context, a conventional MTL paradigm may cause a disconnection between the subsampled raw data and reconstruction–analysis vision, leading to rather limited priori information for neural networks. Here, we present the necessity of reconstruction–analysis collaborative sampling and formulate a new problem of full-stack learning (FSL). The novelty of this work is related to the exploration of a brand new MTL paradigm for a collaborative MR imaging pipeline by starting from observation to human perception to automated machine analysis in a DL manner so that all tasks in the imaging process effectively and efficiently benefit each other in the whole pipeline. As the imaging process progresses from sampling to reconstruction to analysis, the results of each step are suboptimally processed by different devices. To a significant degree, this process can be stepwise mimicked through DL from the scanner with a preset accelerated rate to the reconstructor to the analyst. A novel aspect of our approach is that it combines all task-specific networks into a single network. More precisely, we instantiate FSL by a trial exemplar architecture, a multitask learning network framework, and we simultaneously optimize learnable trajectories, reconstruction vision tasks and analysis vision tasks. This allows obtaining the optimal results of each step to minimize the risks involved in medical imaging. That is, each network performs at its own maximum in the imaging pipeline: (1) a subsampling trajectory fully and coherently expressed by reconstruction–analysis vision; (2) MR images with correct tissue details, plausible high-level semantics, and latent representations of machine vision; and (3) truly trustworthy machine predictions. To implement the FSL approach in a practical manner, reconstruction tasks and analysis tasks are much more efficient and accurate, and their interactions therefore do not have a great divide. This is both reconstruction- and analysis-friendly, and also expected to proactively provide insights into bridging cross-device and cross-domain research from radiology practice, computer vision, and machine learning when AI meets medical big data.

## Results

### Concept of FSL

**Figure 1:**
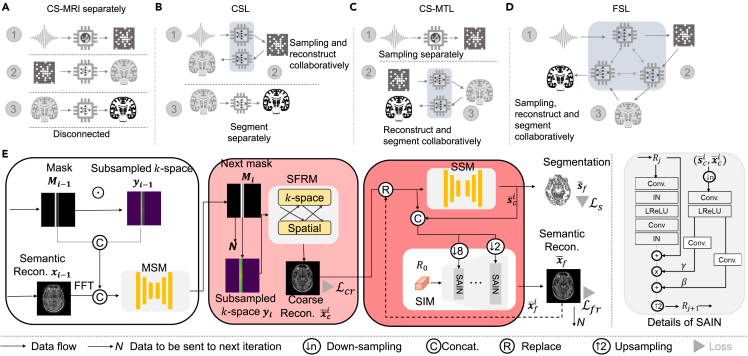
Concept of FSL (A) Illustration of the concept of CS-MRI with sampling, medical image reconstruction and analysis tasks in separate, disconnected workflows. (B) Principle of CSL. (C) CS-MTL, where the reconstruction task is adapted to the analysis task but with sampling locations set by human experience. (D) Principles of FSL with all tasks adapted to each other. (E) Schematic of a trial exemplar FSL consisting of a collaborative multitask network that uses a differentiable approach. Imaging data are individually used by the submodules in a forward manner, and the sampling model collects reconstruction and analysis vision data to predict the next subsampled trajectory.

Conceptually, if data or signals are sufficiently sampled, an imaging workflow can be performed separately (Figure 1A). In CSL, signals are measured in a learnable way so that sampling and imaging can be optimized jointly by pixel references (Figure 1B), which can substantially enhance image reconstruction for examining and thereby improving the imaging results^14^ but poses disadvantages such as the loss of anatomical structures and machine vision representation data, as well as the use of a suboptimal workflow for the imaging pipeline. Multitask learning approaches^17^^,^^18^ have been developed (Figure 1C), wherein joint learning for reconstruction-analysis tasks is responsible for semantically aware inverse problem or analysis-friendly image reconstruction; however, a hand-crafted sampling measurement trajectory is maintained.

As an alternative, we introduce FSL (Figure 1D), which is instantiated by a trial exemplar multitask learning framework (Figure 1E) that optimizes the sampling, reconstruction and segmentation processes jointly in an end-to-end manner. Although our components possess a common design, the way they are assembled is very innovative in three aspects. (1) This method utilizes an easy yet novel end-to-end differentiable approach for this problem to fully explore the mutual influence among the sequential tasks and further improve the performance of each task simultaneously, which not only improves the visual quality of the subsampled MR images via semantic awareness but also boosts the accuracy of clinical tasks. (2) In the downstream task our training strategy can cope with both subsampled inputs and fully sampled inputs without any fine-tuning. (3) We observe that semantic sampling makes the reconstructor generalizable to other datasets with different distributions. We select both the CS-MRI and medical image segmentation tasks to exemplify the potential medical value of FSL.

In the instantiated framework of FSL, an autoencoder to predict next subsampling probability map and sampling operation (Figure S1) to binarize the map in a desired sampling rate is first employed to build a measurement sensing module (MSM) due to its differentiable compressive sampling method. A preset sampling rate trajectory can be predicted progressively by inputting the output to autoencoder, followed by a uniform distribution matrix that achieves Monte Carlo searching and a scaling layer that adjusts prediction to preset sampling rate. Although the processing paradigms for reconstruction-analysis vision have apparent differences, differentially, they are the same learning methods in deep neural networks. Naturally, we are able to utilize the differentiability of these three tasks to conduct measurement and jointly support reconstruction and analysis vision tasks. As a result, spatial-frequency reconstruction module (SFRM) and semantic segmentation module (SSM) (Figure S3) are then employed sequentially to realize the image reconstruction and analysis tasks. We also propose a semantic interaction module (SIM) (Figure 1E) to ensure that both features of reconstruction and segmentation interact sufficiently. SIM outputs a prior semantic reconstructed image, which is sent to MSM that can sense the spatial intense and semantic representation and predicts next subsampled trajectory. This sequential and interactive method can also greatly improve the semantic information contained in the reconstructed images so that they can be efficiently examined by humans and machines.

### Evaluation with 2D trajectories

We first compare our method with three conventional paradigms (Figures 1A–1C, detailed in the Compared methods section), including DL-based CS-MRI, CS-MTL, and CSL. We evaluate our proposed FSL under 5% and 10% sampling rates on the ACDC, OASIS, and NC-ISBI datasets.

**Figure 2:**
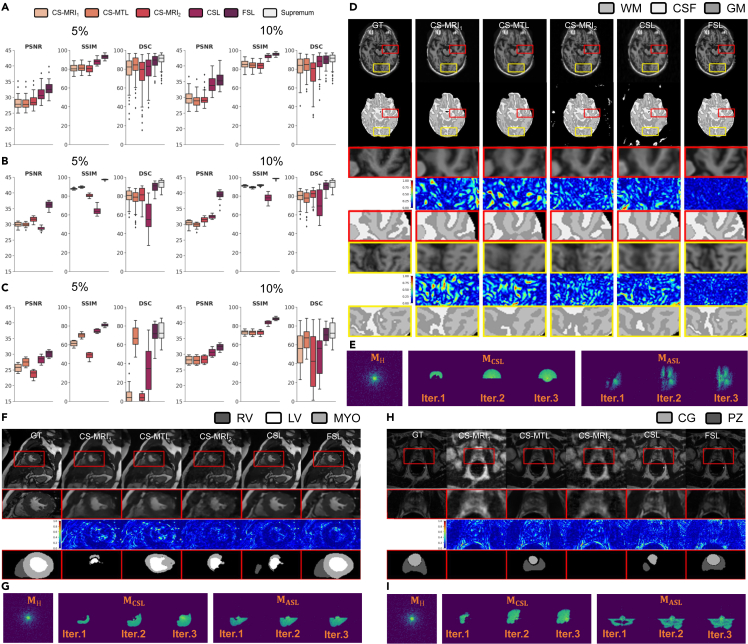
Comparison of FSL with different imaging paradigms based on ACDC, OASIS, and NCI-ISBI datasets for learning 2D sampling trajectories (A–C) Quantitative results with 5% (left) and 10% (right) sampling rates using a variety of paradigms based on ACDC, OASIS and NCI-ISBI datasets respectively. (D) (F) (H) Representative visual results (rate = 5%) of reconstructed images (first row), segmentation maps (second row), zoom-in reconstructed images (third row) in the first ROI, zoom-in reconstructed error maps using NMSE (fourth row) in the first ROI, zoom-in segmentation maps (fifth row) in the first ROI. (D) Zoom-in reconstructed images (sixth row) in the second ROI, zoom-in reconstructed error maps using NMSE (seventh row) in the second ROI, and zoom-in segmentation maps (eighth row) in the second ROI. The ground truth (GT) is shown in the first column, and the images reconstructed using CS-MRI1, CS-MTL, CS-MRI2, CSL and FSL are provided in the second to sixth columns. (E) (G) (I) Sampling k-space amplitude images by corresponding trajectories of hand-tailed (left panel, rate = 5%), CSL learned (middle panel) over three iterations (from left to right) and FSL learned (right panel) over three iterations (from left to right). FSL removes more artifacts, preserves more anatomical details and predicts more accurate segmentations that are in agreement with the ground truth in ROI.

As a conventional DL-based CS-MRI paradigm, CS-MRI_1_ reconstructs images with low performance (Figures 2A–2C) and a limited de-aliasing effect, where significant remaining aliasing artifacts can be observed (Figures 2D, 2F, and 2H). This leads to a decline in the segmentation accuracy of this method (Figures 2A–2C, 2D, 2F, and 2H). On the other hand, CS-MRI_2_ is trained with a dual-domain model to interact information between the spatial and k-space domains, and performs well in terms of the various evaluation metrics (Figures 2A–2C); however, its unreliable reconstructed structure and blurred tissues cause performance degradation on the segmentation task (Figures 2D, 2F, and 2H). Although CSL works quite well in terms of quantitative metrics (Figures 2A–2C) due to its data-adapted trajectories, it still suffers from a distorted structure (Figures 2D, 2F, and 2H), which affects the downstream tasks (Figures 2D, 2F, and 2H). Moreover, CSL suffers from severe overfitting (Figure 2B) on OASIS dataset. Guided by fully sampled segmentation-aware tasks that provide more semantics and machine vision priors, CS-MRI_1_ and CS-MTL reconstruct images that reserve relatively plausible structures (from a qualitative perspective) but with clearly oversmoothed reconstruction details and relatively low performance. By comparison, as FSL utilizes a learnable measurement to integrate reconstruction-analysis vision, it yields better performance (Figures 2A–2C) in subsampling artifact removal, better reliability in maintaining structural information, and more accurate segmentation results (Figures 2D, 2F, and 2H). Even when the sampling rate results in a large loss of raw data, the image structure can still be satisfactorily reserved by FSL. The corresponding sampling trajectories are placed in Figures 2E, 2G, and 2I.

### Evaluation with 1D trajectories

**Figure 3:**
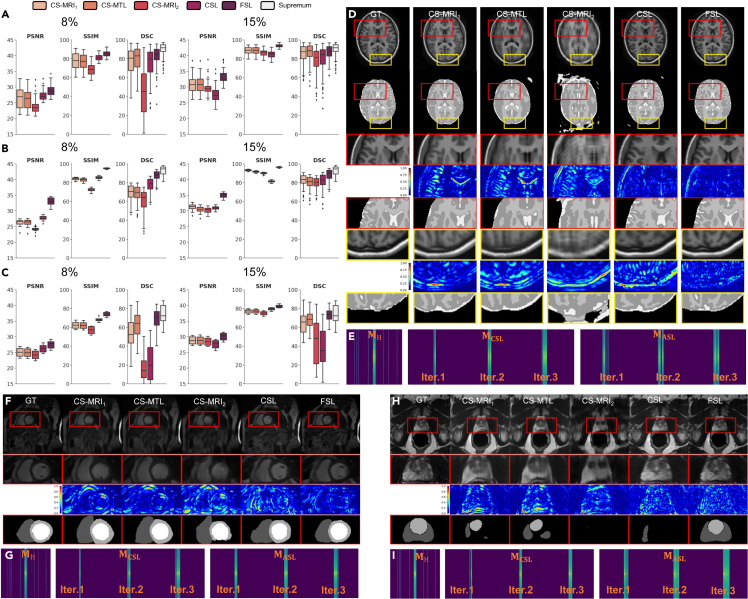
Comparison of FSL with different imaging paradigms based on ACDC, OASIS, and NCI-ISBI datasets for learning 1D sampling trajectories (A–C) Quantitative results with 8% (left) and 15% (right) sampling rates using a variety of paradigms based on ACDC, OASIS and NCI-ISBI datasets respectively. (D) (F) (H) Representative visual results (rate = 5%) of reconstructed images (first row), segmentation maps (second row), zoom-in reconstructed images (third row) in the first ROI, zoom-in reconstructed error maps using NMSE (fourth row) in the first ROI, zoom-in segmentation maps (fifth row) in the first ROI. (D) Zoom-in reconstructed images (sixth row) in the second ROI, zoom-in reconstructed error maps using NMSE (seventh row) in the second ROI, and zoom-in segmentation maps (eighth row) in the second ROI. The ground truth (GT) is shown in the first column, and the images reconstructed using CS-MRI1, CS-MTL, CS-MRI2, CSL, and FSL are provided in the second to sixth columns. (E) (G) (I) Sampling k-space amplitude images by corresponding trajectories of hand-tailed (left panel, rate = 5%), CSL learned (middle panel) over three iterations (from left to right) and FSL learned (right panel) over three iterations (from left to right). FSL outperforms the other methods in removing artifacts, in preserving anatomical details and in predicting accurate segmentation in ROI.

Due to hardware requirements, Cartesian sampling trajectories are considered to validate the performance of the proposed FSL. CS-MRI_1_, CS-MTL and CSL are unable to completely restore reliable structures and predict accurate segmentations (Figures 3D, 3F, and 3H) under different rates in the Cartesian-sampled images. Although CS-MRI_1_ and CS-MTL try to recover more reliable structures, they have lower performance (Figures 3A–3C) and oversmoothed details constrained (Figures 3D, 3F, and 3H) by limited partial measurements (Figures 3E, 3G, and 3I). In contrast, the proposed FSL method performs well in medical segmentation and restores better structures and details.

### Generalization and robustness

In clinic practice, the importance of generalization and robustness of a DL-based imaging model has increased. We conduct detailed experiments and analysis of the differences between the three conventional paradigms and the proposed FSL model on two externally unseen datasets from fastMRI and M&Ms to study the generalization and on noise-corrupted OASIS testing datasets to study the robustness.

**Figure 4:**
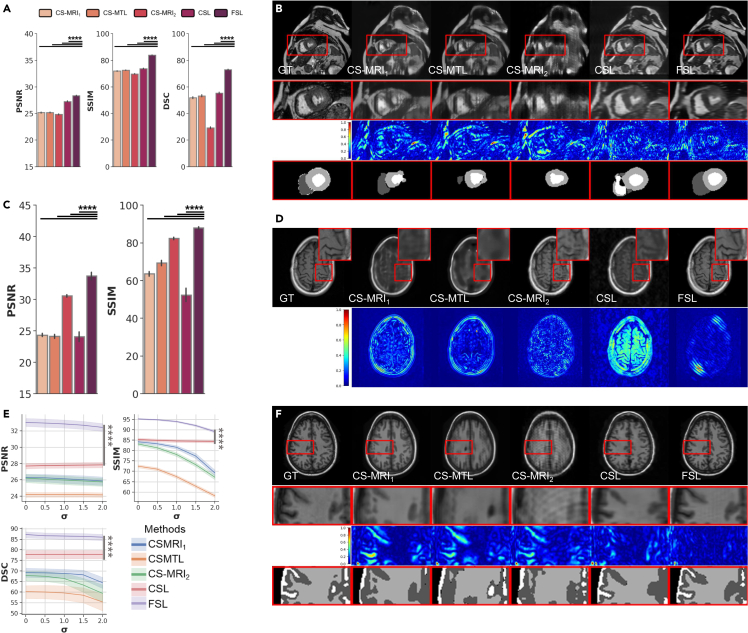
Generalization and robustness comparison of the reconstructed images for different externally unseen testing datasets at different sampling rates in different trajectories with a variety of paradigms (A and B) Quantitative and qualitative generalization results (rate = 8%, 1D trajectories) on M&Ms dataset for evaluating generalization, where representative visual results in (B) are reconstructed images (first row), zoom-in reconstructed images (second row) of ROI, zoom-in reconstructed error maps using NRMSE (third row) of ROI, and zoom-in segmentation maps (fourth row) of ROI. (C and D) Quantitative and qualitative results (rate = 10%, 2D trajectories) on fastMRI T1 test for evaluating generalization, where representative visual results in (D) are reconstructed images (first row), error maps using NRMSE (second row). (E and F) Quantitative and qualitative generalization results on OASIS for evaluating robustness. (E) PSNR (left top), SSIM (right-top), DSC (left bottom) comparison of the robustness of different paradigms at different Rician noise levels σ=0,0.5,1.0,1.5,2.0), where representative visual results in (F) of reconstructed images (first row), zoom-in reconstructed images (second row) of ROI, zoom-in reconstructed error maps using NRMSE (third row) of ROI, zoom-in segmentation maps (fourth row) of ROI. (B), (D), (F) The ground truths are shown in the first column, and the results using CS-MRI1, CS-MTL, CS-MRI2, CSL, and FSL are shown in the second to sixth columns. These samples validate that FSL is robust on sampling, reconstruction and segmentation for images with different noise and generalizable from different scanners.

To investigate the collaboration of FSL, we perform a generalization experiment from externally same imaging modality scans, including M&Ms (cardiac MRI with segmentation reference) at 8% 1D sampling case and fastMRI (Brain MRI without segmentation reference) at 10% 1D sampling case. We find that the results of our method have the best or most competitive scores in comparison with those of other approaches in terms of various metrics (Figures 4A and 4C). In particular, FSL outperforms CSL by a large margin. Moreover, our methods still achieve great performance on the fastMRI and M&Ms datasets in removing subsampled artifacts and recovering more real anatomical structures and textures (Figures 4B and 4D). The results suggest that our method has significant generalization in terms of artifact removal, detailed texture recovery, anatomical structure recovery and segmentation task.

As for robustness, experiments were performed by adding in silico Rician noise to the OASIS testing datasets at 8% 1D sampling case. Silico Rician noise-corrupted subsampled MR images are generated by adding Gaussian noise to the real and imaginary parts. Then we evaluate the performance of the three conventional paradigms and the proposed model in Figure 4E. As the noise level σ increases from 0.5 to 2.0, our method outperforms all other paradigms by a large margin. Moreover, we can observe that conventional imaging paradigms suffer from severe noise distortion (second to fourth columns in Figure 4F) and have unreliable segmentation maps, which is because hand-tailed trajectories or lack of semantics-guidance cannot sample efficient semantic information for supporting anatomical structure. Compared with these methods, FSL can acquire images with more reliable visual quality and details, closing to corresponding groundtruth. The results suggest that FSL interacts semantic representation, supporting trustworthy tissue structure and offsetting the noise corruption.

### Algorithm investigation

#### The effectiveness of coarse- and semantic-reconstruction for segmentation

**Figure 5:**
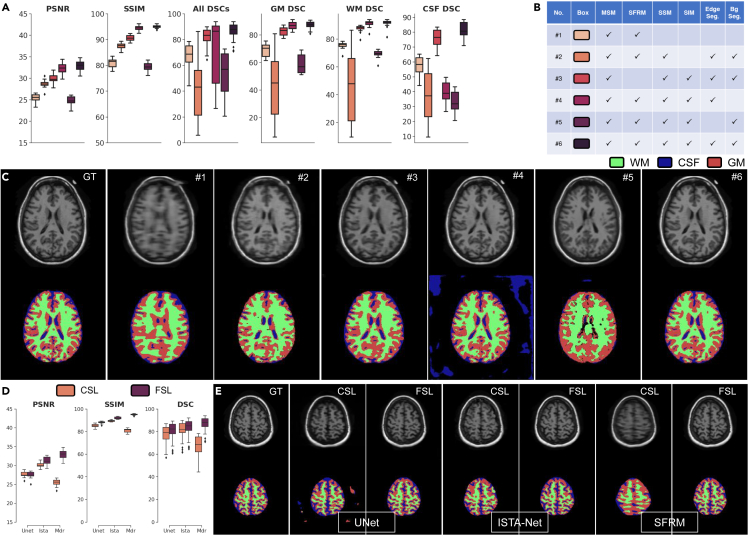
Ablation comparison of FSL with different modules and reconstruction backbones (A), (C) Quantitative and Qualitative ablation results (rate = 8%, 1D trajectories) on the OASIS dataset for evaluating the effectiveness of each module. (A) PSNR (first column), SSIM (second column), DSC (third column), gray matter (GM) DSC (fourth column), white matter (WM) DSC (fifth column) and CSF DSC (sixth column) comparisons of FSL by different combinations (legends placed in (B)). (B) Different ablation combinations of FSL. (C) Representative visual results of reconstructed images (first row), and segmentation maps (second row). The ground truths are shown in the first column, and the results using ablation combination #1 to #6 (explained in (B)) are shown in the second to sixth columns. (D and E), Quantitative and Qualitative results (rate = 8%, 1D trajectories) on OASIS test dataset for evaluating FSL paradigm where representative visual results in (E) of reconstructed images (first row) and segmentation maps (second row) of CSL and FSL on different reconstruction backbones on UNet (second panel), ISTA-Net (third panel) and SFRM (fourth panel). The results of the ablation experiments show that all three modules of FSL play a critical role, and that edge segmentation enhances both reconstruction and segmentation, while background segmentation enables the model to make predictions in the ROI region (A)-(C). FSL can improve the performance of different backbone networks, compared to CSL, and SFRM is well suited for the FSL paradigm (D), (E).

In the interaction between reconstruction and segmentation, a fundamental difficulty in directly segmenting reconstructed images is the negative effects of subsampled artifacts and unreliable tissue of reconstructed images. Here, we use coarse reconstruction to remove subsampled artifacts and use semantic reconstruction to restore reliable anatomical tissue images. In Figures 5A and 5C, we implement FSL without SFRM for coarse-reconstruction (#3 in Figure 5B) and without SIM for semantic-reconstruction (#2 in Figure 5B). The performance drops clearly demonstrate the benefits brought by coarse- and semantic-reconstruction. Moreover, we can observe the method encounters obvious performance drop without FSL. The proposed FSL learns structure-robust features from both pixel and semantic level, which plays an important role in segmentation results.

#### The effectiveness of high-level semantics for reconstruction

Then, we evaluate the effect of the proposed SSM and SIM at different semantic information guided by both segmentation classes of edge and background for reconstruction in Figure 5A. We can observe that the proposed edge segmentation (#4 in Figure 5B) brings a finer image reconstruction while the performance drops (#5 in Figure 5B) demonstrate the benefits brought by edge segmentation. Moreover, with background segmentation in FSL, results possess more anatomical features in ROI (#6 in Figure 5B), where the segmentation results are allowed for more accurate prediction in turn, due to the reliable reconstructed images. As shown in Figure 5C, we visualize the reconstruction results without (#1 in Figure 5B) or with SSM and SIM (#6 in Figure 5B). The method with SSM and SIM achieves visual quality with less subsampled artifacts and reliable bio-tissue structures. Therefore, it can be inferred that anatomical edge segmentation plays a vital role as a bridge between the reconstruction and segmentation processes of the multitask network.

#### The effectiveness of full-stack learning paradigm

To demonstrate the efficiency of our proposed FSL paradigm, we study the FSL extensions on two representative reconstruction backbones, including UNet,^12^ ISTA-Net.^21^ Figures 5D and 5E summarize the numerical and visual results of on OASIS dataset at 10% 2D sampling rate of different reconstruction backbones equipped with different sampling paradigms, from which we observed that our imaging paradigms can cooperate effectively for these reconstruction algorithms and consistency outperforms the CSL paradigms. Note that, FSL with SFRM is still the top-performing FSL algorithm, while ISTA-Net with our FSL paradigm can reach a comparable performance using the same training strategy. These results collectively show the adaptive collaboration (yielded by our FSL) is of great benefit to MR imaging pipeline in terms of both better restoration and segmentation accuracy.

**Figure 6:**
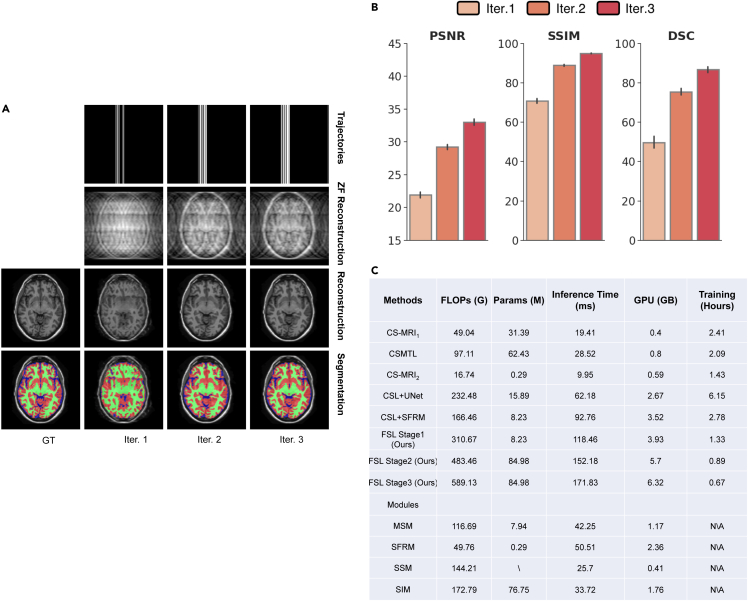
Experimental observation of iteration process and training strategies of FSL (A and B) Qualitative and Quantitative results (rate = 8%, 1D trajectories) on the OASIS dataset for evaluating the effectiveness of each iteration. (A) Representative visual results of sampling trajectories (first row), zero-filling (ZF) reconstructed images (second row), reconstructed images (third row), and predicted segmentation (fourth row). The ground truths are shown in the first column. (B) PSNR (left), SSIM (middle), and DSC (right) comparisons of FSL over three progressively imaging iterations. (C) Model performance of CS-MRI1, CS-MRI2, CS-MTL, CSL and FSL (different training stages and modules). GFLOPs calculation, inference time, training parameters, GPU cost and training time adopt with 240×240 resolution as inputs, batch size = 1. The SSIM and DSC are reported with inference on the OASIS testing dataset (rate = 8%, 1D trajectories).

In addition, to verify progressive collaboration and semantic sampling strategy in our proposed method, how sampling, reconstruction and segmentation results change was analyzed in each sampling inference. The obtained results are shown in Figures 6A and 6B, from which we observe that the performance are improved along iterations simultaneously for both tasks. Better reconstruction would ease the difficulty of segmentation task; anatomical semantic guidance would collaborate reconstruction in turn; Knowledge-prior k-space trajectories can be obtained by inferencing MR images rich in semantics.

#### Evaluation on real-clinic multicoil raw data

**Figure 7:**
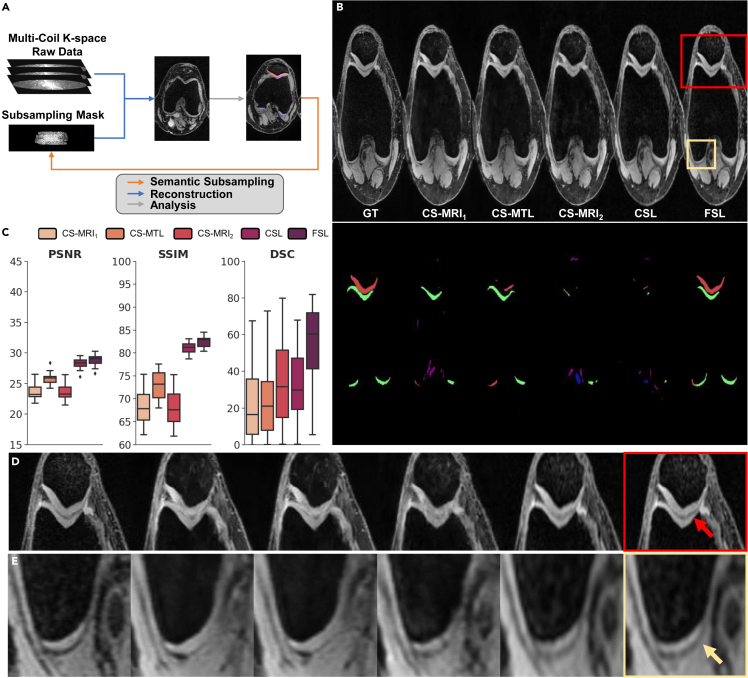
Comparison of FSL with different imaging paradigms on clinical SKMTEA dataset (A) FSL applied to clinical data with multi-coil raw data. (B and C) Qualitative and quantitative results (ratio = 10%, 2D trajectories) using a variety of paradigms, respectively. (D and E) Enlarged ROIs, corresponding one-to-one with each column in (B).

To assess the performance of multitask on FSL paradigm for real scenarios, experiments were conducted on SKMTEA^22^ with real-clinical multicoil raw knee data, reconstructed images, and segmentation labels. We evaluated our FSL and other methods on SKMTEA at 10% sampling ratio. The experimental results are depicted in Figure 7. It is observed that: (1) The proposed FSL achieves substantial improvements in PSNR, SSIM, and DSC when compared with CSL; (2) From a visual perspective, FSL demonstrates superior reconstruction and segmentation results in the ROI area. These findings suggest that integrating three tasks of sampling, reconstruction, and segmentation into our FSL effectively benefits each other’s performance in real-world conditions. The proposed FSL shows a stable performance and strong generalization capability in real-world scenarios.

#### Investigation of transfer learning and few-shot learning

**Figure 8:**
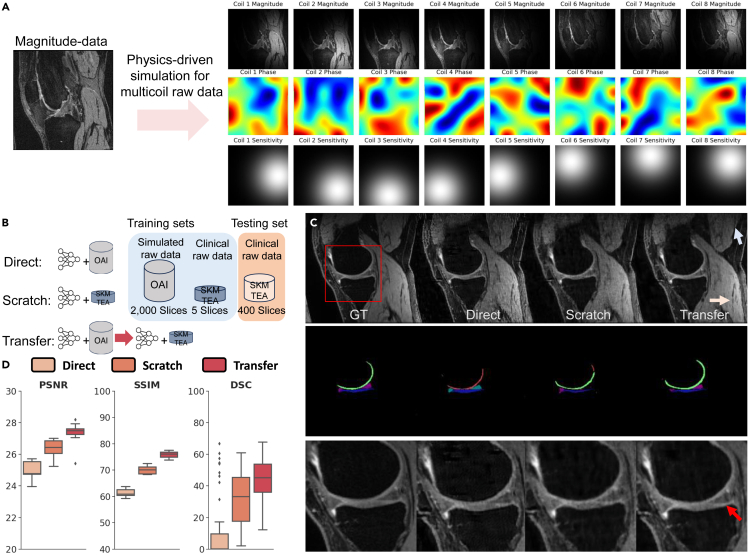
Investigation in transfer learning of FSL (A) Physics-driven multicoil simulation on images with only magnitude values, accompanied by segmentation labels. (B) Three methods for evaluating transfer learning and few-shot learning. Direct: trained on OAI simulated raw data; Scratch: trained on clinical SKMTEA data with only 5 slices; Transfer: network weights trained on OAI simulated raw data used as initial values, followed by training on clinical SKMTEA data with only 5 slices. In the testing stage, all methods were tested on clinical SKMTEA data for sampling, reconstruction, and segmentation. (C and D) Qualitative and quantitative results (ratio = 10%, 2D trajectories) on clinical SKMTEA dataset.

Multitask data is very difficult to collect. To overcome the lack of multitask data for reconstruction and segmentation, we conducted experiments with 2D trajectory at 10% sampling ratio. We first pre-trained FSL model on 2,000 physics-driven simulation knee magnitude images with segmentation labels from OAI,^23^ as depicted in Figure 8A; then, transfer learning (finetuning in our experiments) and few-shot learning (5 slices in our experiments) were applied to the pre-trained model on the real-world clinical SKMTEA.^22^ The finetuned model was noted as ‘Transfer’. As demonstrated in Figure 8B, two other compared methods were conducted: ‘Direct’, which was trained on OAI simulated data; and ‘Scratch’, which was trained on clinical SKMTEA data using only 5 slices. In the testing stage, all methods were evaluated on the clinical SKMTEA data. Experimental results in Figure 8 show that ‘Transfer’ outperforms ‘Direct’ and ‘Scratch’ in both reconstruction and segmentation performance qualitatively (Figure 8C) and quantitatively (Figure 8D). The results demonstrate that our FSL can leverage the knowledge acquired from one dataset to enhance performance on other datasets with limited labeled data through transfer learning and few-shot learning, thereby addressing the challenges arising from the lack of multitask datasets.

## Discussion

Our data suggest that reconstruction and analysis vision largely regard their measurement positions as an efficient collaboration that is capable of enhancing the representation of reconstruction-analysis vision in the whole imaging pipeline. Selecting a correct position with regard to arranging the measurements of reconstruction-analysis vision and collaborating the three tasks (measurement, reconstruction, medical segmentation) are associated with higher performance. Through the three comparative studies in our experiments, we find that FSL achieves performance that is better than or comparable to that of other methods despite the lack of semantic information. We therefore confirm that the key paradigm of our proposal, i.e., FSL, contributes to achieving such high performance. In conventional CS-MRI paradigms, powerful models are designed to improve visual perception. Although such paradigms improve perception, our experiments and previous works suggest that some information is lost in the representation of reconstruction vision, while more is lost in analysis vision with a data-independent sampling trajectory. From the results, CSL approaches illustrate that even if sampling is associated with image texture details, without semantic association, k-space sampling trajectories cannot be fully optimized. In our experiments, CS-MTL shows that even with a segmentation task guide, reconstruction fails without a sampling mapping of texture and semantic information.

We presented an FSL approach, which is a full-stack framework for reconstruction and analysis tasks with learnable observation in a medical imaging workflow. We have demonstrated the full-stack collaborative training of a measuring inference method in the challenging imaging task of CS-MRI and the subsequent segmentation process. Furthermore, we have showcased quantitative and qualitative superiorities of FSL over the conventional MRI paradigms on externally unseen datasets and noise-corrupted datasets. Our work serves as the first step toward the implementation of next-generation imaging pipeline methods in medical imaging workflows. It can be applied to multitask research in many fields, such as video coding^24^ and radio astronomical observations,^25^ allowing for the collaboration of up- and downstream tasks and mastery over compressed sensing data with the theoretical and empirical guarantees of visually intermediate checks and a human–machine-friendly dataflow. FSL represents a targeted evolution of our previous work toward all-in-one focused deployment. Although we focus on a segmentation task for the presented case study, FSL is highly adaptable to a variety of medical imaging analysis workflows employing different network architectures, datasets and more. To date, no work has been published on the role of a learnable observative FSL model—an area where if feasible, its use as a collaborator for reconstruction-analysis vision would be an incredible breakthrough in our mastery over imaging pipelines.

In conclusion, we presented a novel FSL paradigm for medical image reconstruction and analysis tasks that are integrated by a learnable observative model on medical imaging data, and we showcase the paradigm in a clinically relevant real-life case study. Further research and development will enable a visual- and machine-friendly sequential design of our framework, the validation of our findings on sequential imaging, and the further widespread utilization of FSL techniques in healthcare and beyond. Importantly, the possibility of contributing to changing the way we collaborate with machines is a very exciting proposition.

### Limitations of the study

In FSL, although we have achieved multi-task success on raw data, there is still a lack of multitask datasets that support both reconstruction and analysis vision in evaluating the performance of sampling learning methods. Specifically, common medical image datasets are mainly designed for image reconstruction tasks and lack high-level labels, while previous attempts toward sampling-based learning focused on pixel supervision and could not restore machine semantics. This paper presents the first attempt toward the use of compressed sampling to support reconstruction and segmentation vision simultaneously and provides a preliminary and new exploration direction that imposes higher demands on data. In this paper, we choose mainly those MRI datasets for two reasons. First, they have important research value in functional analysis and diagnostics. More importantly, these parts of humans have been well studied in the medical imaging community, and there are rich related segmentation and detection models that can provide labels for high-level tasks. Nevertheless, in future studies, due to the lack of suitable datasets, it is compromised to use the frequency data simulated from the spatial images as the raw data, which might not be an accurate simulation of the acquisition process. Hence, we call for the release of more expert-level annotations of medical diagnostics for raw data, and how to study unsupervised semantics for machine tasks is also our future research direction.

## STAR★Methods

### Key resources table

**Table:** 

REAGENT or RESOURCE	SOURCE	IDENTIFIER
**Deposited data**		

Automated Cardiac Diagnosis Challenge (ACDC) dataset	University Hospital of Dijon, France	https://www.creatis.insa-lyon.fr/Challenge/acdc/
the Open Access Series of Imaging Studies (OASIS) dataset	Washington University, USA	http://www.oasis-brains.org/#data
NCI-ISBI 2013 Challenge - Automated Segmentation of Prostate Structures dataset	The National Cancer Institute, USA	https://wiki.cancerimagingarchive.net/display/public/nci-isbi+2013+challenge+-+automated+segmentation+of+prostate+structures
the Multi-Centre, Multi-Vendor and Multi-Disease Cardiac Segmentation (M\&Ms) dataset	Universitat de Barcelona, Spain	https://www.ub.edu/mnms/
fastMRI dataset	New York University, USA	https://fastmri.org/
The SKM-TEA dataset	Stanford AIMI Center, USA	https://github.com/StanfordMIMI/skm-tea
The Osteoarthritis Initiative (OAI) dataset	National Institutes of Health, USA	https://nda.nih.gov/oai/

**Software and algorithms**		

Our proposed FSL	This paper	https://github.com/wangzhiwen-scu/FSL

**Other**		

Quadro RTX 8000	NVIDIA Corporation, USA	https://www.nvidia.com

### Resource availability

#### Lead contact

Further information and requests for resources and reagents should be directed to and will be fulfilled by the lead contact, Yi Zhang (yzhang@scu.edu.cn).

#### Materials availability

This study did not generate new unique reagents.

#### Data and code availability

• All data are available in the main text. We analyzed multiple published MRI datasets throughout the evaluations. These data are available as follows: (1) Automated Cardiac Diagnosis Challenge (ACDC) dataset (https://www.creatis.insa-lyon.fr/Challenge/acdc/ ); (2) the Open Access Series of Imaging Studies (OASIS) dataset (http://www.oasis-brains.org/#data); (3) NCI-ISBI 2013 Challenge - Automated Segmentation of Prostate Structures dataset https://wiki.cancerimagingarchive.net/display/public/nci-isbi+2013+challenge+-+automated+segmentation+of+prostate+structures; (4) the Multi-Centre, Multi-Vendor and Multi-Disease Cardiac Segmentation (M&Ms) dataset (https://www.ub.edu/mnms/); (5) fastMRI dataset (https://fastmri.org/); (6) The SKM-TEA dataset (https://github.com/StanfordMIMI/skm-tea); and (7) The Osteoarthritis Initiative (OAI) (https://nda.nih.gov/oai/).
• The FSL model was developed using standard libraries in the open-source platform PyTorch 1.7.1. The source codes of FSL, several representative pre-trained models as well as some example images for testing are publicly accessible via: https://github.com/wangzhiwen-scu/FSL. The code base is made available for non-commercial and academic purposes. The datasets and the code are publicly available as of the date of publication.
• Any additional information required to reanalyze the data reported in this paper is available from the lead contact upon request (yzhang@scu.edu.cn).

### Method details

#### Datasets

We start the exploration of our FSL framework on cardiac, brain and prostate MR images, as they are usually highly structured, share unified tissue components, provide strong priors for reconstruction-analysis tasks, and lead to a very important research area of medical image analysis. We train FSL by using five training datasets, ACDC, OASIS, NCI-ISBI, OAI and SKMTEA, and evaluate it on themselves’ testing datasets, respectively. In addition, we added Racian noise to test the robustness of the model and tested the generalization of the model on fastMRI and M&Ms datasets. There are seven datasets in the multi-task experiments: ACDC (cardiac MRI, for training and testing), OASIS (brain MRI for training and testing), NCI-ISBI (prostate MRI for training and testing), M&Ms (cardiac MRI for testing), fastMRI (brain MRI for testing), OAI (knee MRI for multicoil data training), and SKMTEA (knee MRI for multicoil data testing). Since there is no large design for both MR image reconstruction and segmentation, subsampled k-space data are obtained by executing preset subsampled trajectories on the segmentation benchmark. Here are details for each dataset.

• ACDC.^26^ We collect the cardiac T1-weighted MRI scans from Automated Cardiac Diagnosis Challenge (ACDC), including 50 subjects for training, and 50 subjects for evaluation. The cardiac tissues of each patient are labeled with three types, including Left-Ventricle (LV), Right Ventricle (RV) and Myocardium (MYO).
• OASIS.^27^ The brain dataset from the Open Access Series of Imaging Studies (OASIS) is used to evaluate the proposed method. Each patient aged 18 to 96 is provided with T1-weighted MRI modalities. Each scan had approximately 180 slices with expert annotations for cortical gray matter (GM), white matter (WM), and cerebrospinal fluid in the extracerebral space (CSF). We collect 20 patients for training, and 20 patients for evaluation.
• NCI-ISBI.^28^ The abdominal T2-weighted MRI dataset from the NCI-ISBI 2013 Challenge - Automated Segmentation of Prostate Structures, comprises of 20 scans for training and 10 scans for evaluation, where each scan had approximately 30 slices with expert annotations, including central gland (CG) and peripheral zone (PZ).
• M&Ms.^29^ We test our FSL model for generalization on an external cardiac MRI dataset from the Multi-Centre, Multi-Vendor and Multi-Disease Cardiac Segmentation (M&Ms). The training dataset is acquired on two scanners at multi-centers and consists of 150 patients. The dataset of each patient is provided with T1 modalities where three organs were annotated, including LV, RV and MYO. In our experiment, all patients are used for testing. M&Ms and ACDC are slightly different w.r.t their scanning parameter settings.
• FastMRI.^30^ It is a brain benchmark dataset for MRI reconstruction. Since no ground truth is available for dataset segmentation, we test our FSL model only for reconstruction generalization on the external fastMRI Brain subset. The brain subset data are acquired on 3T and 1.5T scanners and consist of 6970 samples, and the test dataset consists of 860 samples. The subset of each patient is provided with four MRI modalities, including FLAIR, T1 weighted, T1 weighted with contrast agent (T1 POST) and T2 weighted. In our experiments, we choose randomly 80 emulated^31^ single-coil (ESC) brain T1-weighted MRI scans for evaluation. FastMRI and OASIS are greatly different w.r.t their scanning parameter settings.
• SKMTEA.^22^ The SKMTEA multitask dataset contains multicoil k-space raw data, reconstructed images, and expert-derived segmentation labels from 155 knee qMRI scans. Each scan has two echoes, and we chose the first one for our experiments, where $k_{x}\times k_{y}\times k_{z}=512\times 512\times 160$ for each k-space volume with voxel spacing [0.3125,0.3125,0.8] mm. Constrained by the storage cost of k-space data, we randomly chose 12 scans for training and 8 scans for testing from SKMTEA. For *Sec. Evaluation on real-clinic multicoil raw data*, note that only the middle 300 slices $k_{y}\times k_{z}$ were used in the transverse plane. For *Sec. Investigation of transfer learning and few-show learning*, the middle 80 slices $k_{x}\times k_{y}$ were used in the sagittal plane. The selection of the middle slices was to include most of the labels. Expert-derived segmentation labels in SKMTEA are provided: patellar cartilage, femoral cartilage, lateral tibial cartilage, medial tibial cartilage, lateral meniscus, and medial meniscus.
• OAI.^23^ The Osteoarthritis Initiative (OAI) dataset contains 240 double-echo DESS knee MRI scans. Each image data volume size is 384×384×160 and it’s voxel spacing is 0.3646×0.3646×0.7 mm. Only the reconstructed sagittal images and expert-devived segmentation labels are provided. The segmentation labels include femoral cartilage, medial tibial cartilage, lateral tibial cartilage, patellar cartilage, lateral meniscus, and medial meniscus. 20 scans were selected randomly and simulated into multicoil raw data for training.

#### Evaluation metrics

We evaluate our algorithm against the above state-of-the-art MRI methods in terms of image quality and segmentation accuracy on open-source datasets and use the peak-signal-to-noise ratio (PSNR), structural similarity index (SSIM) as reconstruction evaluation metrics, and Dice similarity coefficient (DSC) as segmentation evaluation metrics.

#### Compressed sensing MRI

MR imaging can be formulated as the following inverse problem:

(Equation 1) y=Ax+n

where $x\in C^{M\times M}$ stands for a 2D MR image, $y\in C^{M\times N}$, represents fully-sampled measurements in k-space (i.e., Fourier space), n stands for measured error and system noise, and A=FM is the measurement process, where F is Fourier transform matrix and $M\in {\left\{1\right\}}^{M\times N}$ is a binary sampling trajectory. In compressed sensing (CS) MRI, only a partial observation $M\in {\left\{0,1\right\}}^{M\times N}$ is implemented by a certain sampling rate α, which equals the matrix size of MRI divided by observed measurement.

Recent advances in compressed sensing (CS) magnetic resonance imaging (MRI) focus on deep learning based MRI reconstruction from undersampled data, effectively reducing artifacts and improving image quality.^32^ A pioneer work is to perform CS-MRI reconstruction with convolutional neural network (CNN).^2^ Similarly, in,^33^ the authors adopt Generative Adversarial Networks (GANs) to further refine the reconstructed images and improve the perceptual quality and diagnostic viability. Different from conventional network architectures, Transformer models^34^ were introduced to capture long-range dependencies and hierarchical structures in image data and showed improved reconstruction of intricate anatomical structures. In,^35^ Graph Neural Networks (GNNs) serve as a powerful tool to model implicit relations in image data, delivering perceptible improvements in reconstructing images from undersampled k-space data. However, these methods suffer from huge computational and GPU memory consumption. In contrast, the dual-domain model, MD-Recon-Net,^36^ pays more attention to the fidelity of k-space data and utilizes frequency-domain and image-domain information in a lightweight yet high-performance manner. Since FSL requires multiple iterations of different tasks, leading to a multiplicative increase in computational load and GPU memory usage, the lightweight dual-domain model is suitable for our FSL paradigm.

#### Trial exemplar architecture of FSL

We first give an overview of our **FSL** approach (Figure 1D). Figure 1E illustrates the proposed trial exemplar architecture of FSL method. Our framework combines segmentation and reconstruction with trajectory learning, which involves anatomical structural sampling. The main modules of the proposed framework, including the measurement sensing module (**MSM**), spatial-frequency reconstruction module (**SFRM**), semantic segmentation (**SSM**) and semantic interaction module (**SIM**), are first sequentially described in detail. Then, other issues regarding the proposed architecture, especially those related the training strategy and loss function, are presented.

#### Measurement sensing module

For MR image reconstruction, the sampling trajectory is commonly empirically hand-tailored to avoid subsampling and aliasing, e.g., radial, Cartesian, or spatial Boolean matrices. However, a fixed trajectory ignores the fact that different anatomical structure and reconstruction model may be suitable for different subsampling trajectories, which will miss the semantic measurements of trapping into the local optimum. In response to this challenge, we proposed MSM to progressively sampling the efficiently semantic measurements (Figure S1). Given a fully-sampled measurement y and sampling trajectory $M_{i}$ (a center preset sampling $M_{0}$ as the initial trajectory), in the i th iteration, the process can be written as:

(Equation 2) $$y_{i-1}=M_{i-1}⊙y$$

where ⊙ is the Hadamard product. The MSM outputs a sampling trajectory $M_{i}$ as a partial observation, which is progressively predicted by MSM with the i-1th iteration subsampling reconstructed image ${\bar{y}}_{i-1}$.

The goal of the MSM is to predict the sampling pattern for specific issue datasets in the k-space:

(Equation 3) $$M_{i}=MSM\left({\bar{x}}_{i-1},M_{i-1}\right)\text{.}$$

To predict a probabilistic observation matrix in the k-space, we adopt a similar architecture^15^ for our MSM. Inspired by LOUPE^14^ and SeqMRI,^15^ the architecture and details of the MSM are shown in Figure S1. Here, we show two different predicted trajectories for specific prior tissue knowledge in Figures 2E, 2G, 3E, 3G, and 3I.

We conduct two different sampling trajectory cases experiments for sampling, reconstruction and segmentation. The specific details are outlined below.

##### Task1: 2D trajectory (Gaussian-like) learning

Constrained by equipment, a Gaussian-like trajectory is still calculated in this paper to demonstrate the high performance of our approach. The top-n largest values in $T_{MC}$ (Figure S1) are replaced by Boolean values to produce the final sampling pattern $M_{i}$, and n is chosen according to the predetermined sampling rate α, where n=α·M·N. Accordingly, the Booleanizing operation can be written as:

(Equation 4) $$\left(M_{kl}\right)=\left\{ \begin{array}{cc} 1, & \text{if}\;{\left(T_{MC}\right)}_{kl}\;\text{is}\;\text{in}\;\text{top}-n\\ 0 & \text{otherwise}. \end{array}\right.$$

##### Task2: 1D trajectory (Cartesian) learning

Cartesian trajectory learning is a common sampling method for structural MRI in conventional spin echo (CSE or SE) and fast spin echo (FSE) pulse sequences, but this approach suffers from slow data acquisition. The fractional number of excitations (NEX) accelerates MRI by reducing the number of encoded phases.

In this case, a subsampling trajectory $M_{i}$ is searched in a 2D sampling space $T_{MC}^{M\times N}$ (Figure S1). First, the weights $T_{MC}$ of each phase encoding are obtained by summing the frequency encoding, $\sum_{0}^{N}T_{MC}^{M\times N}$ . Then, the binary process is the same as that the without constrained trajectory. The process above can be written defined as:

(Equation 5) $$\left(M_{k}\right)=\left\{ \begin{array}{cc} 1, & \text{if}\;{\left(T_{MC}\right)}_{k}\;\text{is}\;\text{in}\;\text{top}-n\\ 0 & \text{otherwise}. \end{array}\right.$$

Finally, we extend the binary result to a 2D matrix (along the frequency encoding axis). Since we do not have the labels for sampling pattern learning, we propose to merge the MSM into the FSDM, SSM and SIM described below. When the cascaded network converges, the output $M_{i}$ of the MSM will be optimal.

#### Spatial-frequency reconstruction module

Recently, extensive network models have been proposed for MRI reconstruction, and in this work, we carefully modify a Spatial-Frequency Reconstruction Module (SFRM). Letting SFRM(·) denotes the SFRM, $F^{-1}$ is the inverse Fourier transform matrix, $y_{i}$ is the sampling measurement, and $x_{i}=F^{-1}y_{i}$ is the zero-filling image, and the coarse reconstructed image $\bar{x}$ can be obtained in the i th iteration as:

(Equation 6) $${\bar{x}}_{c}^{i}=SFRM\left(x_{i},y_{i}\right)$$

The SFRM branches of the FSL are trained as a coarse reconstructing integral with the objective function defined as:

(Equation 7) $$L_{cr}=\|{\bar{x}}_{c}^{i}-x_{gt}{\|}_{2}^{2}$$

where $\|\cdot {\|}_{2}^{2}$ is a L2 loss used to measure the similarity between the reconstructed image and the corresponding label. The architecture of the SFRM adopts the interactive representation infusing between k-space and image domains as the backbone,^36^ and this kind of backbone has demonstrated competitive performance in artifact reduction for ours FSL.

#### Semantic segmentation module

In recent years, prior structural or edge knowledge has been exploited by gradient guidance in deep learning-based methods, which is a regularization function derived from structural priors. Model-based methods sometimes draw prior structures from limited data in a time-consuming way. On the other hand, current deep learning-based methods cannot exploit artificially issued structural information in the k-space. Furthermore, both of the above approaches cannot provide a feedback structure prior for entering the k-space. In this work, we utilize hand-crafted prior segmentation with structural knowledge, which is realized by a Semantic Segmentation Module (SSM) of a supervised neural network, to exploit the optimal structural priors in the k-space to improve the reconstruction and segmentation robustness of the model and prevent overfitting:

(Equation 8) $${\bar{s}}_{c}^{i}=SSM\left({\bar{x}}_{c}^{i}\right)$$

where $s_{c}^{i}$ is the coarse segmentation in the i th iteration. Recently, many networks have been proposed for automatic tissue segmentation 8,12. Since the UNet-like architecture has demonstrated excellent medical image segmentation performance, in this section, we also choose the same network structure shown in Figure S2 as our SSM for simplicity.

#### Semantic interaction module

As mentioned above, the interaction between the imaging pipeline i.e., sampling, reconstruction, and segmentation is inadequate in MSM, SFRM and SSM, which will result in limited performance in the whole pipeline, since it only communicates information in a gradient manner. Therefore, we take a further step towards exploring all stages interaction learning in the pipeline. Specifically, we propose a novel Semantic Interaction Module (SIM) (see the right panel in Figure 1E), which adopts spatially-adaptive normalization layers to effectively integrate the semantic label into the interaction process from low to high image scales. As a result, the model can learn to guide the SFRM process to adjust semantic information and further benefit both the reconstruction and segmentation tasks. In the ith iteration, we take the coarse reconstruction ${\bar{x}}_{c}^{i}$ and coarse segmentation ${\bar{s}}_{c}^{i}$ as input to SIM to get a fine reconstruction, ${\bar{x}}_{f}^{i}$, which will be input again to SSM to get a fine segmentation, ${\bar{s}}_{f}^{i}$. This fine reconstruction ${\bar{x}}_{f}^{i}$ will be inputted to MSM for predicting the next sampling trajectory in the next iteration. We use SIM to obtain a fine reconstructed image ${\bar{x}}_{f}^{i}$:

(Equation 9) $${\bar{x}}_{f}^{i}=SIM\left({\bar{x}}_{c}^{i},{\bar{s}}_{c}^{i}\right)$$

(Equation 10) $${\bar{s}}_{f}^{i}=SSM\left({\bar{x}}_{f}^{i}\right)$$

The FSL samples P times to progressively refine the reconstruction and segmentation: ${\bar{x}}^{i},{\bar{s}}^{i}=\left({\bar{x}}_{f}^{i},{\bar{s}}_{f}^{i}\right)$. $\left({\bar{x}}_{f}^{P},{\bar{s}}_{f}^{P}\right)$ are the final refined reconstructed image and semantic segmentation maps, respectively. We use L2 loss functions to make SIM convergence:

(Equation 11) $$L_{fr}=\|{\bar{x}}_{f}-x_{gt}{\|}_{2}^{2}$$

Cross-entropy loss is utilized for the SSM:

(Equation 12) $$L_{seg}=-\sum_{k}s_{gt}^{k}\ln\;{\bar{s}}_{f}^{k}$$

for K tissue class labels, where $s_{gt}^{k}$ is the target label and $\bar{s_{f}^{k}}$ is the softmax segmentation probability for the $k^{th}$ class. In addition, under unstable observations, the model needs to take the upstream and downstream tasks into account, but the characteristics of the learning process for the upstream and downstream tasks are very different, and this requires a loss function to build a bridge between the two. In a recent work, anatomical edge^37^ provides a bridge between low-level and high-level vision. While the L1 loss in Equation 11 enforces holistic supervision from the fully sampled ground-truth MR image, the boundaries between the key tissues (e.g., cortical gray matter, white matter and ventricles) of brains may be easily obscured as they are distorted under a certain sampling ratio. The use of the L1 loss alone is likely to fail to restore true tissue boundaries from artifacts. Thus, inspired by SROBB,^37^ we propose organ edge detection and foreground/background extraction as extra segmentation labels to explore strong semantic mapping in the k-space, generate semantically reconstructed results and improve the accuracy of downstream tasks. The process of label creation is shown in Figure S4. The organ edge and background enforce the FSL to explore more semantic details with fewer subsampled artifacts and generate latent representations for downstream tasks with respect to MR images.

#### Compared methods

We use a trail exemplar FSL and conduct an ablation-like study as our basic experiment to demonstrate that a) semantic exploration in the k-space is crucial for high-quality image and anatomy reservation and b) the guidance of representative semantic features is the key to providing a sampling- and learning-based reconstruction task. With respect to a), we train a Unet^12^ as baseline CS-MRI_1_. In addition, for further guidance by semantic representation, we follow Liu et al.^20^ and pretrain the segmentation networks as CS-MTL that are pretrained on the fully sampled image, which essentially is FSL without a sampler, i.e., joint reconstruction and segmentation with fixed trajectories – with the guidance of semantic information but without semantic exploration in the k-space. With regard to b), we train a joint learning network for trajectory optimizing and reconstruction, i.e., seqMRI^15^ as CSL, without the guidance of semantic features. Moreover, we also introduce a state-of-the-art model MD-Recon-Net^36^ as CS-MRI_2_ to demonstrate that an excellent model might fail when the sampling trajectory, distribution of the dataset, and reconstructor are mismatched.

**CS-MRI**_**1**_: We build a variant of our FSL as a baseline that learns reconstruction with fixed trajectories. We train this model using the same training strategy as that of our FSL method.

**CS-MTL**: Inspired by Liu et al.,^20^ We build a variant of our FSL as a baseline that learns reconstruction and segmentation simultaneously with fixed trajectories and a pretrained segmentation network. We train this model using the same training settings as that of our FSL method.

**CS-MRI**_**2**_: The MRI dual-domain reconstruction network MD-Recon-Net is a recently proposed dual-domain reconstruction with fixed trajectories. It contains two parallel and interactive branches that simultaneously operate on the k-space and spatial-domain data.

**CSL**: seqMRI is a recently proposed sampling pattern learning model driven by reconstruction.

For **CS-MRI**_**1**_, **CS-MRI**_**2**_, and **CSL**, we train them with paired MR images for reconstruction test. In testing, the inputs are the reconstructed images in the pretrained segmentation network same with **CS-MTL** and **FSL**. In our next experiments, we empirically study the behaviors of FSL. We pay special attention to what may contribute to the model’s mutual benefits among the three subnetworks.

#### Training strategy

We train the proposed framework on the three described tasks. Considering their diversity under unstable measurements, it is difficult to jointly train the whole network synchronously. The proposed model adopts the following progressive training strategy:

• Stage1: We first fix the pretrained SSM and train the MSM and SFRM using L2 loss and CE loss for 3,000 iterations.
• Stage2: We then fix MSM, SFRM and SSM and train the SIM using the L2 loss and CE loss for 3,000 iterations.
• Stage3: Finally, we jointly optimize both MSM and SIM by minimizing the L2 loss and CE loss for 3,000 iterations.

We demonstrate that adopting such a training strategy guarantees that the learned sampling pattern can acquire as much useful information as possible for the subsequent reconstruction and segmentation tasks. More specifically, the proposed FSL can be easily adapted to different clinical tasks, and we can substitute the SSM with any other task network. Our approach not only facilitates the training effort while utilizing the ReconNet to fit clinical tasks and keeping the SSM performing accurately for subsampled MR images but also enables the sampler to learn more clinically useful features from the k-space data.

#### Implementation details

Then, the hybrid loss function for the proposed joint learning network is formulated as:

(Equation 13) $$L=L_{cr}+\lambda_{1}L_{sseg}+\lambda_{2}L_{fr}$$

where $\lambda_{1}$, $\lambda_{2}$ are the weighting parameters used to balance the tradeoff between the two components. The number of the iteration i for FSL is set to be 3. All implementations are based on PyTorch. All models are trained using one Quadro RTX 8000 GPU, and the batch size is set to 12. Uniform random initialization is used for the sampler and Xavier initialization for SFRM and SSM. The ADAM optimizer is adopted with an initial learning rate of $5e^{-4}$. $\lambda_{1}$ and $\lambda_{2}$ are empirically set to $1e^{-1}$ and $1e^{10}$, respectively. We use a batch size of 12 and apply random rotation-based data augmentation within ±10°. The whole training process takes approximately 0.5 days on the above GPU card. To preprocess the data, we used PyTorch real-time data augmentation. To save computation, all images were truncated to 240×240 in k-space from their different original size. We simulate the sampling process by using trajectories.
