# Supplementary material for: Promoting fast MR imaging pipeline by full-stack AI
Source: iScience. 2023 Dec 2;27(1):108608. doi: 10.1016/j.isci.2023.108608 (PMC10762466; doi:10.1016/j.isci.2023.108608)
Supplement: Document S1. Figures S1–S4 [file mmc1.pdf]

iScience, Volume *27*

Supplemental information

Promoting fast MR imaging pipeline by full-stack AI

Zhiwen Wang, Bowen Li, Hui Yu, Zhongzhou Zhang, Maosong Ran, Wenjun Xia, Ziyuan Yang, Jingfeng Lu, Hu Chen, Jiliu Zhou, Hongming Shan, and Yi Zhang

Supplementary Information

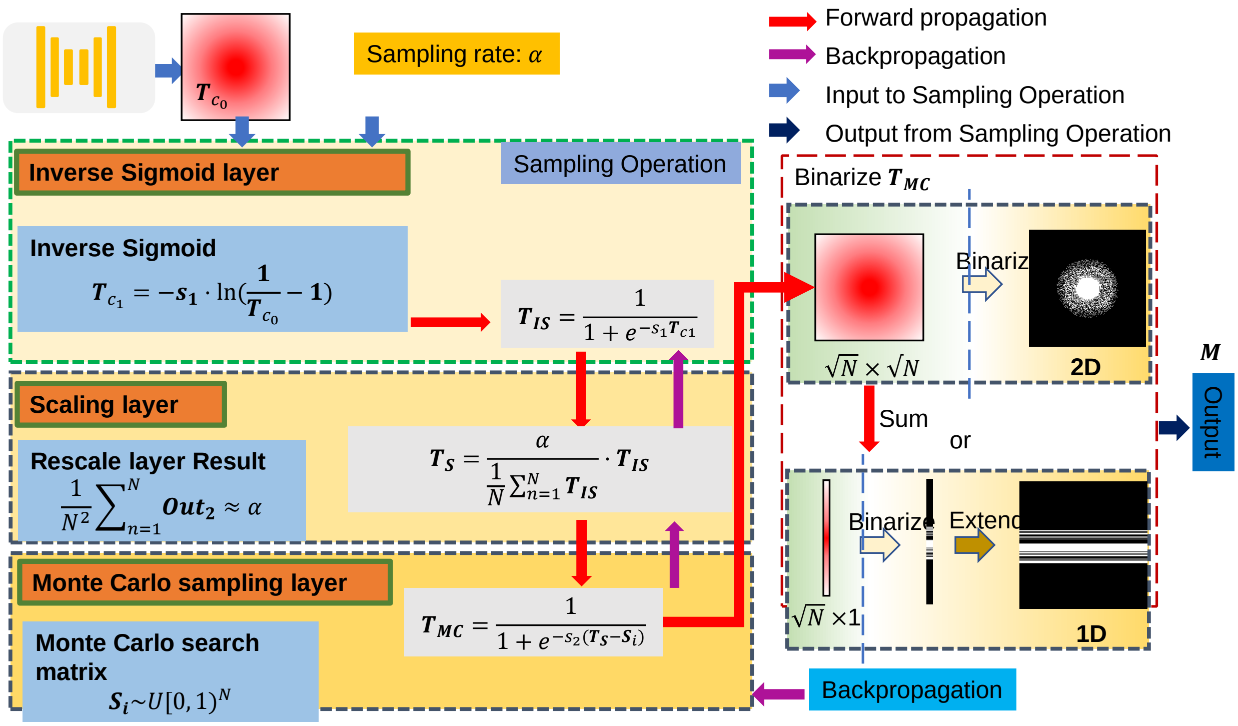

Figure S1: The detailed architecture of the MSM, related to STAR Methods. The post-process of the proposed **MSM** is operated by three layers, i.e., inverse sigmoid layer, scaling layer and Monte Carlo sampling layer. First, sampling map $\boldsymbol{T}_{c_0}$ is predicted by an encoder-decoder on the interval [0,1). Then, $\boldsymbol{T}_{c_0}$ is mapped to a real number set with an inverse sigmoid function. The parameter layer performs a nonlinear transform by using a parametrized sigmoid function. Next, we rescale the mean of the input matrix to a predetermined sampling rate α in the scaling layer. In the last layer, Monte Carlo sampling is approximatively realized, and the parametrized sigmoid function is also applied to the result.

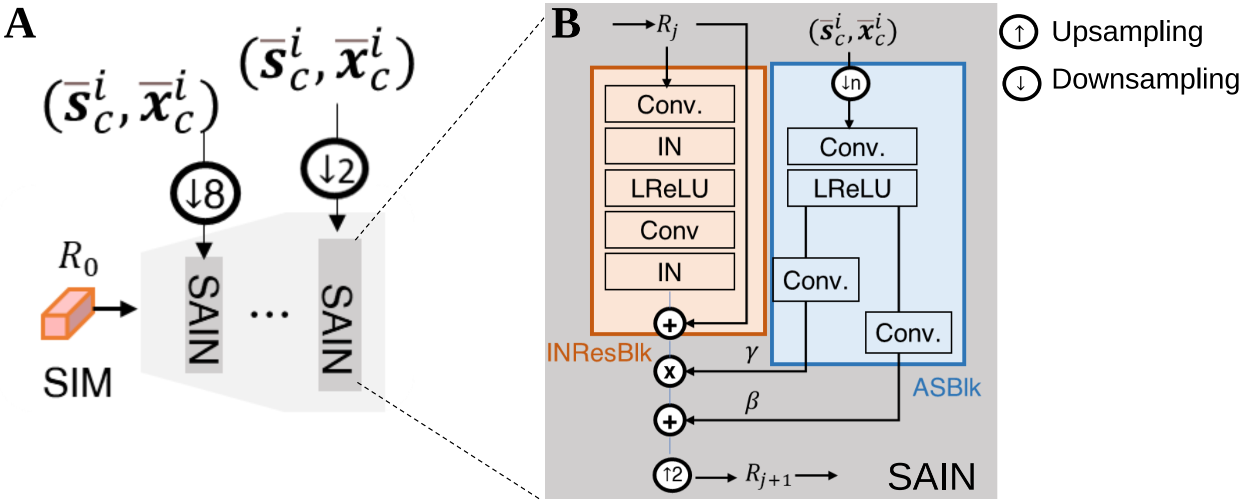

Figure S2: The detailed architecture of the Semantic Segmentation Module (SIM) and Semantic-aware Adaptive Instance Normalization (SAIN), related to STAR Methods. **A**) an overview of Semantic Interaction Module (SIM), which sets the coarse reconstruction and segmentation by a decoder as semantic prior. We propose a simple yet effective interacting method called Semantic-aware Adaptive Instance Normalization (SAIN). We utilize the SFRM and SSM to get a coarse pair result $(\bar{\boldsymbol{s}}_c^i, \bar{\boldsymbol{x}}_c^i)$ of segmentation and reconstruction, respectively. The coarse reconstruction and segmentation are sent to SAIN to provide prior semantics for refining reconstructed imaging. **B**) The details of SAIN module, which consists of instance normalization residual block (INResBlk) with inputting latent representations, and adaptive semantic block (ASBlk) with inputting a coarse pair result. Specifically, we take SAIN module in the i-th layer of SIM for example. Let $R_j \in \mathbb{R}^{C_j H_j W_j}$ be the representations in the j-th residual blocks, where C_j, H_j, W_j denote the channel, height, weight of representations R_j, respectively. As depicted in (B), we first operate the input representations R_j by the INResBlk. Then, the ASBlk extracts the semantic mean γ^j and variance β^j from both $(\bar{\boldsymbol{s}}_c^j, \bar{\boldsymbol{x}}_c^j)$ to predict latent semantic maps. The new representation values R_{j+1} in the i-th layer is computed by: $R_{j+1} = \text{UPBlk}(\gamma^j(R_j - \mu(R_j))/\sigma(R_j) + \beta^j)$ where UPBlk is the upsamling convolutional block (UPBlk). SAIN helps the network to capture the visual semantic representation from the semantic segmentation maps and inject it into the reconstructed image, which can be sensing in the next sampling stage. Therefore, the reconstructed ROI region are more reliable in anatomical structure.

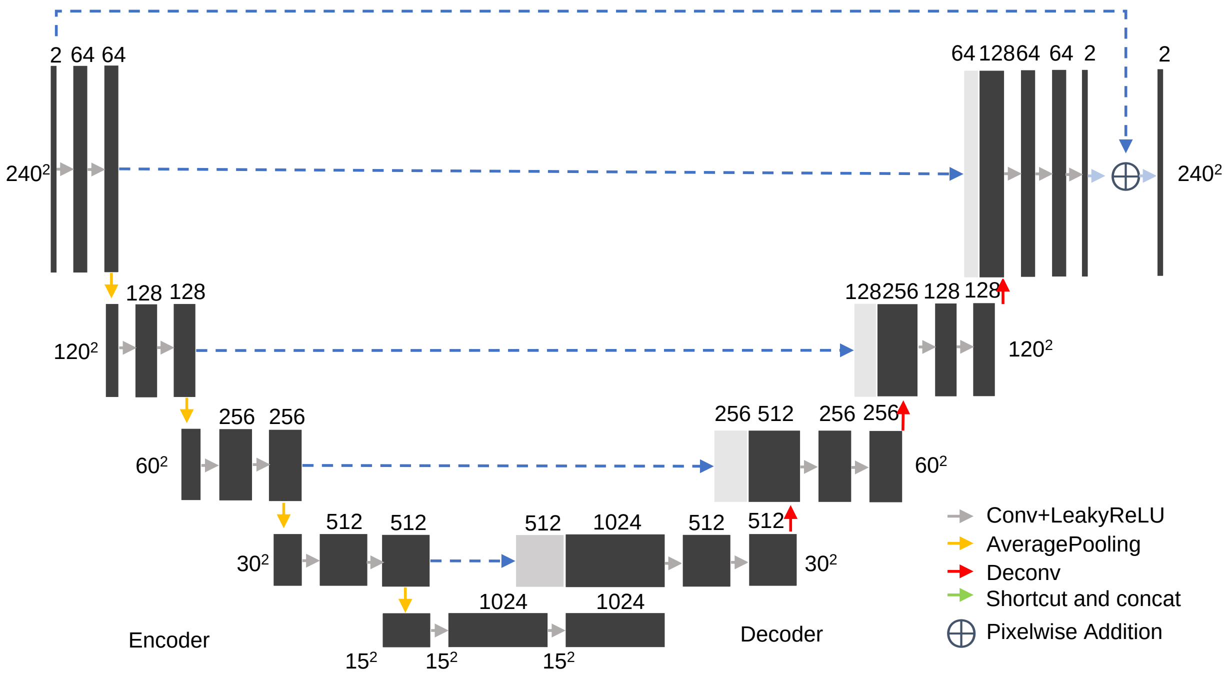

Figure S3: An encoder-decoder architecture, related to STAR Methods. In the encoder part, the Unet obtains an data as input and uses convolution and pooling to compress the data into a latent space at a different scale. In the decoder part, Unet attempts to use deconvolution to predict the pixel value probability map by decoding the hidden information from the latent space. The use of a skip connection between the encoding part and the decoding part can help the model to better preserve detailed feature information for anatomical context exploration.

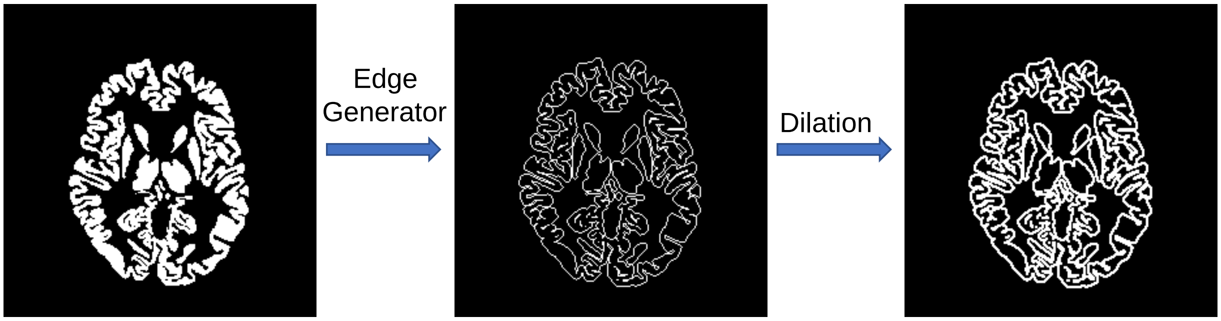

Figure S4: The construction of an anatomical boundary label, related to STAR Methods.
